# Family and Physician Perspectives: Unassailable, a p53 and PIK3CA Mutant Glioblastoma in a Child

**DOI:** 10.3390/curroncol33010008

**Published:** 2025-12-23

**Authors:** Mary-Pat Schlosser, Lea Stelter, Mike Stelter

**Affiliations:** 1 Stollery Children’s Hospital, Edmonton, AB T6G 2B7, Canada; 2 Patient Partner, Edmonton, AB T6G 2B7, Canada

**Keywords:** pediatric neuro-oncology, central nervous system tumours, high-grade glioma, pediatric radiotherapy, chemotherapy

## Abstract

High-grade gliomas in children continue to have a dismal prognosis. This case is of a child with a diffuse pediatric-type high-grade glioma H3 and IDH-wildtype, treated with surgery, radiation, chemotherapy, and a targeted agent without success. Physician and family perspectives are also presented. Options for treatment and research participation are limited, but there is a strong desire from patients and families to access new treatment modalities and take part in clinical trials. Treating teams and families remain hopeful that in the future, tumours like the one described in this report will be treated with much greater success.

## Simple Summary

Pediatric high-grade gliomas are difficult-to-treat brain tumours with poor prognosis. Despite attempts to treat one patient’s high-grade glioma with surgery, radiation, and chemotherapy, we were unsuccessful. It is the perspective of his parents and treating physician that these types of tumours warrant further research and that patients should have access to clinical trial participation and novel therapies. There is tremendous hope that the prognosis for difficult-to-treat pediatric brain tumours will one day be much improved.

## 1. Clinical Case

At the age of 4 years, after new right-sided seizures, magnetic resonance imaging (MRI) showed a 5 × 4 × 3 cm mass in Ben’s left posterior temporal–occipital region. Ben’s name is used intentionally with permission from his parents. On MRI his tumour had significant surrounding vasogenic edema and was heterogeneously T2 hyperintense and T1 hypointense, with ill-defined areas of restricted diffusion, and a central area of necrosis with post-contrast rim enhancement. Ben underwent gross total resection of the tumour and recovered quickly from surgery. Pathology revealed a glioblastoma, IDH-wildtype [1], now classified as a diffuse pediatric-type high-grade glioma H3 and IDH-wildtype [2]. The tumour was negative for MGMT and TERT promoter methylation and negative for mutations in BRAF, H3, and TP53. Thirty-four days after surgery, he started photon radiation therapy, 54 Gy in 30 fractions with concurrent temozolomide [3]. He did not have access to proton radiation therapy. Aside from fatigue, he tolerated radiation well. He continued with temozolomide and lomustine maintenance post-radiation [4] and required one dose reduction for pancytopenia.

Eight months after initial resection, Ben had a new enhancing lesion seen in the left temporal lobe on MRI within the previous radiation field. A second resection was performed, but this time post-operative imaging showed tiny nodular enhancing lesions in the resection cavity. Pathology was again consistent with diffuse pediatric-type high-grade glioma, H3-wildtype and IDH-wildtype with microvascular proliferation, palisading necrosis, and abundant bizarre mitotic figures. Additional clinical molecular pathology now demonstrated p53 p.R196Wfs*11 and PIK3CA p.G1049R variants. Germline TP53 testing confirmed the presence of a likely pathogenic variant in the patient. Ben’s parents had consented for him to participate in a research study, the PRecision Oncology For Young peopLE (PROFYLE) study [5], to perform further genomic analysis of his tumour. This revealed a number of additional alterations including deletions, mutations, amplifications, and increased ribonucleic acid (RNA) expression, a few of which were thought to have possible treatment targets, though none with a strong level of support. A follow-up MRI less than 1 month after his second surgery demonstrated a large enhancing mass, this time with evidence of subependymal spread. Weeks later, prompted by new headache and fatigue, another MRI showed further increase in the size of the recurrent lesions.

Ben’s parents wanted to pursue ongoing therapy and consider participation in clinical trials. Unfortunately, no curative treatment options were available, nor were there any accessible suitable research trials. However, based on the high homologous recombination deficiency score demonstrated in research genomic analysis, it was decided that a poly(ADP-ribose) polymerase (PARP) inhibitor could be considered if access could be obtained. Ben was able to start niraparib 100 mg PO daily, but after 2 weeks he had to stop due to thrombocytopenia. The intention was to restart at a reduced dose once platelets recovered, or consider alternate therapy. Unfortunately, he progressed symptomatically, and imaging confirmed aggressive progression of the tumour with new areas of hemorrhage.

Without tolerable treatment options, Ben’s parents opted to trial complementary and alternative therapy including cannabidiol (CBD) oil, hoping for both symptomatic and treatment benefits. They made a trip to Disneyland and returned for care that focused on symptom management, including reirradiation, and activities to enhance his quality of life. Ben died from his disease at the age of 6 years, almost 18 months after initial diagnosis.

## 2. Physician’s Perspective

In training, I remember learning that the leading cause of cancer death in children had recently become central nervous system (CNS) tumours, despite the overall higher incidence of hematologic malignancy [6]. This struck me as a powerful reflection on the improvement in care for children with leukemia, and a strong indication of the need for similar work to now be carried out for CNS tumours. Ben’s story reminds us of the experience of patients and families in these difficult scenarios. And it reminds us of the immense possibility for advancement.

I choose to work in pediatric neuro-oncology because I believe it is an area on the cusp of significant improvement in treatment and outcomes. I am excited by the development of targeted therapies and applicable small molecules [7,8,9]; the application of immunotherapy to this patient group [10]; increased access to improved treatment modalities, such as proton radiation to reduce long-term side effects; and the exploration of innovative techniques such as Tumour Treating Fields [11,12].

It is our turn within the field of pediatric oncology to heavily increase focus on pediatric CNS tumours. It is our patients’ turn to benefit from the diligent work of clinicians and researchers to advance care to the degree we have seen in hematologic malignancies.

Ben and his family faced a horrible scenario: an aggressive tumour, surgery, radiation, chemotherapy, a germline variant in TP53, and a progressive and ultimately fatal outcome. It was critical for us to offer to Ben and his family treatment, supportive care, participation in available research, germline TP53 testing and counselling, and palliative care. I wish Ben had survived his cancer, and I believe in the future more children in his circumstance will.

Ben had a wonderful, thoughtful, and playful personality. When I saw him, his voice often sounded as if he were on the brink of laughter. His warm and caring demeanour was a reflection of the compassion and empathy of his parents. Ben and his parents were driven, too. They never gave up; they always focused on what was right for Ben. Even now, his parents direct The Ben Stelter Foundation. Their organization funds pediatric brain tumour research, supports the care and quality of life for children and families with cancer, and is advancing access to proton radiation therapy for patients in Canada. Their work inspires us to do better.

## 3. Family Perspective

When Ben was diagnosed, we were hopeful that the different treatments he would receive would help, and that the cancer would go away and stay away. What we didn’t realize at the time was that the standard treatment for his tumour (surgery, radiation, and chemotherapy), and its outcome, hadn’t really changed in decades, and that outcomes remained very poor. It felt like nothing had changed and there didn’t seem to be any real progress forward in the world of pediatric high-grade glioma. It was surprising and disheartening to think that there was no significant research, or new treatments, on the horizon for pediatric brain cancers like Ben’s.

As parents we inquired about clinical trials, different forms of chemotherapy, or any additional testing that could be done to try to stop or even slow down the progression of his tumour growth. There are no proton therapy treatment centres in Canada, and access to proton treatment outside of Canada is not always supported or feasible [13]. Knowing more about it now, we wonder if it could have been helpful. When dealing with such an aggressive cancer, his quality of life in the 18 months that he lived through it was the most important factor in our minds as his parents. We still wanted him to enjoy life and to be able to partake in fun activities with his sisters and us just as he had before he was diagnosed.

There are many things we wish could have happened during Ben’s diagnosis and ongoing treatments. We wish that there were more information and options available to families. From the outside looking in, it seemed as though many other types of pediatric cancer had improved treatments and options for research trial participation. When it came to high-grade glioma, it felt as though there was only one path to go. Not even to save Ben or cure the cancer but just to keep him alive a little bit longer. We would have been very eager to take part in any options for different types of research or clinical trials, or at least have the choice of them. The one research project we were grateful to get to participate in was the PRecision Oncology For Young peopLE (PROFYLE) study for which we consented to sending away samples of his tumour. As parents, we would love to know more from that project: what it means for the future of Ben’s cancer, or even the impact on future patients from having the knowledge learned from Ben’s tumour genetic analysis. Even though Ben passed away, we still have so much hope for the future care of children with pediatric brain tumours and the progress that can be made in the years to come. We are hopeful for advancements on the horizon and what that will mean for future families and children like Ben.

Ben had a passion for his favourite hockey team, the Edmonton Oilers. There wasn’t a day that went by that he wasn’t talking about them or trying to imitate the players and play hockey like them in our home. While Ben went for radiation, his healthcare team quickly learned what a huge fan he was and every single day they would greet him with some sort of conversation about the Oilers or the game the night before. This helped immensely with being able to motivate Ben to go to his appointments and he was excited to go see his friends at radiation every single day during his treatment. He was positive and happy and didn’t complain or cry about having to have chemotherapy or to lay down for radiation. The level of care that everyone gave and that he received daily was outstanding. I truly believe it helped his overall mental well-being, and it made the overall process feel a lot less stressful and heavy. The genuine love and care the entire nursing team always gave to Ben really carried him through and it truly made a difference in our lives and how we were able to remain hopeful and positive.

## 4. Discussion

High-grade gliomas are some of the most difficult-to-treat tumours in children. They come with significant challenges including diffuse infiltrative nature, limiting surgical resection [14]; genetic tumour heterogeneity and clonal evolution at relapse [15]; and leptomeningeal dissemination at progression [16], all contributing to dismal prognosis [17]. Additional challenges to improve outcomes for these children include the high cost of research to target a rare tumour, no transformational progress in trials to help focus efforts, the necessity for collaborative research given the relatively small and dispersed population, the direct and indirect costs incurred by families to participate in clinical trials, and evermore precarious funding to support research for these diagnoses. Despite this, hope for improvement in treatment options and access to research participation remains strong. The willingness of children and families to take part in trials should encourage us to continue our attempts to tackle this sometimes seemingly impossible diagnosis. Clinicians and scientists must continue the frequently thankless-feeling advocacy for support for improvements in care for children with brain tumours. The involvement of patient and family advocates in pediatric cancer research is also a critical step to move forward. Also, it is so important for us as clinicians and families to identify what contributes to a child’s quality of life and to do our best to support care that takes this into account.

## Data Availability

No new data were created or analyzed in this study. Data sharing is not applicable to this article.

## Abbreviations

The following abbreviations are used in this manuscript:

MRI	Magnetic Resonance Imaging
PROFYLE	PRecision Oncology For Young peopLE
RNA	Ribonucleic acid
PARP	Poly (ADP-ribose) polymerase
CBD	Cannabidiol
CNS	Central Nervous System

## References

[1] Louis D.N., International Agency for Research on Cancer (2016). WHO Classification of Tumours of the Central Nervous System.

[2] WHO Classification of Tumours Editorial Board (2021). Central Nervous System Tumours.

[3] Bennett J., Erker C., Lafay-Cousin L., Ramaswamy V., Hukin J., Vanan M.I., Cheng S., Coltin H., Fonseca A., Johnston D. (2020). Canadian Pediatric Neuro-Oncology Standards of Practice. Front. Oncol..

[4] Jakacki R.I., Cohen K.J., Buxton A., Krailo M.D., Burger P.C., Rosenblum M.K., Brat D.J., Hamilton R.L., Eckel S.P., Zhou T. (2016). Phase 2 study of concurrent radiotherapy and temozolomide followed by temozolomide and lomustine in the treatment of children with high-grade glioma: A report of the Children’s Oncology Group ACNS0423 study. Neuro-Oncol..

[5] PROFYLE. https://www.profyle.ca/.

[6] Siegel D.A., Richardson L.C., Henley S.J., Wilson R.J., Dowling N.F., Weir H.K., Tai E.W., Buchanan Lunsford N. (2020). Pediatric cancer mortality and survival in the United States, 2001–2016. Cancer.

[7] Hargrave D.R., Moreno L., Broniscer A., Bouffet E., Aerts I., Andre N., Shen W.P.V., Bertozzi-Salamon A.I., Cohen K.J., Dunkel I.J. (2018). Dabrafenib in pediatric patients with *BRAF* V600–positive high-grade glioma (HGG). J. Clin. Oncol..

[8] Hargrave D.R., Terashima K., Hara J., Kordes U.R., Upadhyaya S.A., Sahm F., Bouffet E., Packer R.J., Witt O., Sandalic L. (2023). Phase II Trial of Dabrafenib Plus Trametinib in Relapsed/Refractory *BRAF* V600–Mutant Pediatric High-Grade Glioma. J. Clin. Oncol..

[9] Rosenberg T., Yeo K.K., Mauguen A., Alexandrescu S., Prabhu S.P., Tsai J.W., Malinowski S., Joshirao M., Parikh K., Farouk Sait S. (2022). Upfront molecular targeted therapy for the treatment of BRAF-mutant pediatric high-grade glioma. Neuro-Oncol..

[10] Karbhari N., Frechette K.M., Burns T.C., Parney I.F., Campian J.L., Breen W.G., Sener U.T., Lehrer E.J. (2025). Immunotherapy for High-Grade Gliomas. Cancers.

[11] Green A.L., Mulcahy Levy J.M., Vibhakar R., Hemenway M., Madden J., Foreman N., Dorris K. (2017). Tumor treating fields in pediatric high-grade glioma. Child’s Nerv. Syst..

[12] Stupp R., Taillibert S., Kanner A., Read W., Steinberg D., Lhermitte B., Toms S., Idbaih A., Ahluwalia M.S., Fink K. (2017). Effect of Tumor-Treating Fields Plus Maintenance Temozolomide vs Maintenance Temozolomide Alone on Survival in Patients with Glioblastoma: A Randomized Clinical Trial. JAMA.

[13] Safavi A.H., Freeman C., Cheng S., Patel S., Mitera G., Kundapur V., Rutledge R., Tsang D.S. (2023). Proton Therapy in Canada: Toward Universal Access and Health Equity with a Publicly Funded Facility. Int. J. Radiat. Oncol. Biol. Phys..

[14] Wisoff J.H., Boyett J.M., Berger M.S., Brant C., Li H., Yates A.J., McGuire-Cullen P., Turski P.A., Sutton L.N., Allen J.C. (1998). Current neurosurgical management and the impact of the extent of resection in the treatment of malignant gliomas of childhood: A report of the Children’s Cancer Group trial no. CCG-945. J. Neurosurg..

[15] Hoffman M., Gillmor A.H., Kunz D.J., Johnston M.J., Nikolic A., Narta K., Zarrei M., King J., Ellestad K., Dang N.H. (2019). Intratumoral Genetic and Functional Heterogeneity in Pediatric Glioblastoma. Cancer Res..

[16] Wagner S., Benesch M., Berthold F., Gnekow A.K., Rutkowski S., Strater R., Warmuth-Metz M., Kortmann R.D., Pietsch T., Wolff J.E. (2006). Secondary dissemination in children with high-grade malignant gliomas and diffuse intrinsic pontine gliomas. Br. J. Cancer.

[17] Vanan M.I., Eisenstat D.D. (2014). Management of high-grade gliomas in the pediatric patient: Past, present, and future. Neurooncol. Pract..

